# Supplementary material for: Impact of food supplements on early child development in children with moderate acute malnutrition: A randomised 2 x 2 x 3 factorial trial in Burkina Faso
Source: PLoS Med. 2020 Dec 23;17(12):e1003442. doi: 10.1371/journal.pmed.1003442 (PMC7757806; doi:10.1371/journal.pmed.1003442)
Supplement: S1 Table — Table A in S1 Table: effects of supplementary foods on MDAT z-scores at end of intervention (12 weeks, n = 1,548): unadjusted analysis. Table B in S1 Table: effects of supplementary foods on MDAT z-scores at end of intervention (12 weeks) in PP population (n = 1,548). Table C in S1 Table: effects of supplementary foods on MDAT z-scores at end of intervention (12 weeks, n = 1,548): sensitivity analysis using minimum values for data imputation. Table D in S1 Table: effects of supplementary foods on MDAT z-scores at end of intervention (12 weeks, n = 1,548): sensitivity analysis using maximum values for data imputation. Table E in S1 Table: effects of supplementary foods on MDAT z-scores after intervention (24 weeks, n = 1,503): unadjusted analysis. Table F in S1 Table: effects of supplementary foods on MDAT z-scores after intervention (24 weeks) in PP population (n = 1,382). MDAT, Malawi Development Assessment Tool; PP, per protocol. (DOCX) [file pmed.1003442.s001.docx]

**Table A in S1 Table: Effects of supplementary foods on MDAT z-scores at end of intervention (12-wk, n=1,548): unadjusted analysis**

|  | **Matrix:**  **LNS versus CSB** | **Soy quality:**  **Isolate versus dehulled** | **Milk protein:** | |
| --- | --- | --- | --- | --- |
|  |  |  | **20% versus 0%** | **50% versus 0%** |
| **Gross motor domain** | 0.03 (-0.05; 0.11) | 0.001 (-0.08; 0.08) | 0.01 (-0.09; 0.11) | 0.01 (-0.08; 0.11) |
| **Fine motor domain** | -0.06 (-0.15; 0.03) | 0.02 (-0.07; 0.11) | 0.02 (-0.09; 0.13) ^a^  In LNS: -0.12 (-0.27; 0.03)  In CSB: 0.16 (0.01; 0.32) | 0.02 (-0.08; 0.13) ^a^  In LNS: -0.06 (-0.21; 0.09)  In CSB: 0.11 (-0.04; 0.26) |
| **Language domain** | -0.01 (-0.10; 0.07) | 0.07 (-0.01; 0.16) | 0.09 (-0.01; 0.19) | 0.11 (0.01; 0.21) |

Data are mean difference (95% CI) based on intention-to-treat population. Linear mixed models adjusted for baseline measure of the outcome (random effects: site). ITT = intention-to-treat. ^a^ Interaction between matrix and milk protein: p =0.07.

**Table B in S1 Table: Effects of supplementary foods on MDAT z-scores at end of intervention (12-wk) in PP population (n=1,429)**

|  | **Matrix:**  **LNS versus CSB** | **Soy quality:**  **Isolate versus dehulled** | **Milk protein:** | |
| --- | --- | --- | --- | --- |
|  |  |  | **20% versus 0%** | **50% versus 0%** |
| **Gross motor domain** | 0.03 (-0.05; 0.11) | -0.01 (-0.09; 0.07) | 0.004 (-0.10; 0.10) | 0.002 (-0.10; 0.10) |
| **Fine motor domain** | -0.03 (-0.12; 0.06) | 0.02 (-0.07; 0.11) | 0.06 (-0.05; 0.17) ^a^  In LNS: -0.07 (-0.23; 0.08)  In CSB: 0.20 (0.04; 0.36) | 0.03 (-0.07; 0.14) ^a^  In LNS: -0.02 (-0.17; 0.13)  In CSB: 0.09 (-0.06; 0.25) |
| **Language domain** | -0.002 (-0.08; 0.09) | 0.06 (-0.02; 0.14) | 0.10 (-0.002; 0.21) | 0.13 (0.03; 0.23) |

Data are mean difference (95% CI) based on intention-to-treat population. Linear mixed models adjusted for baseline measure of the outcome, WHZ, MUAC, HAZ, age, sex, month of inclusion (random effects: site). PP = per protocol. ^a^ Interaction between matrix and milk protein: p =0.07.

**Table C in S1 Table: Effects of supplementary foods on MDAT z-scores at end of intervention (12 wk, n=1,548): Sensitivity analysis using minimum values for data imputation**

|  | **Matrix:**  **LNS versus CSB** | **Soy quality:**  **Isolate versus dehulled** | **Milk protein:** | |
| --- | --- | --- | --- | --- |
|  |  |  | **20% versus 0%** | **50% versus 0%** |
| **Gross motor domain** | 0.05 (-0.03 to 0.13) | -0.01 (-0.09 to 0.07) | 0.01 (-0.10 to 0.10) | 0.01 (-0.08 to 0.10) |
| **Fine motor domain** | -0.04 (-0.12 to 0.05) | 0.01 (-0.07 to 0.10) | 0.01 (-0.09 to 0.12) ^a^  In LNS: -0.14 (-0.29 to 0.01)  In CSB: 0.16 (0.02 to 0.31) | 0.03 (-0.08 to 0.13) ^a^  In LNS: -0.06 (-0.20 to 0.09)  In CSB: 0.11 (-0.04 to 0.25) |
| **Language domain** | 0.004 (-0.08 to 0.08) | 0.07 (-0.01 to 0.15) | 0.09 (-0.01 to 0.18) | 0.11 (0.01; 0.21) |

Data are mean difference (95% CI) based on intention-to-treat population. Linear mixed models adjusted for baseline measure of the outcome, WHZ, MUAC, HAZ, age, sex, month of inclusion (random effects: site). ITT = intention-to-treat. *Interaction between matrix and milk protein: p =0.049.

**Table D in S1 Table: Effects of supplementary foods on MDAT z-scores at end of intervention (12 wk, n=1,548): Sensitivity analysis using maximum values for data imputation**

|  | **Matrix:**  **LNS versus CSB** | **Soy quality:**  **Isolate versus dehulled** | **Milk protein:** | |
| --- | --- | --- | --- | --- |
|  |  |  | **20% versus 0%** | **50% versus 0%** |
| **Gross motor domain** | 0.05 (-0.03 to 0.13) | -0.02 (-0.09 to 0.06) | 0.002 (-0.09 to 0.10) | 0.01 (-0.09 to 0.10) |
| **Fine motor domain** | -0.06 (-0.16 to 0.04) | 0.01 (-0.09 to 0.11) | 0.01 (-0.11 to 0.14 ) ^a^  In LNS: -0.14 (-0.31 to 0.03)  In CSB: 0.16 (-0.01 to 0.34) | 0.02 (-0.10 to 0.14) ^a^  In LNS: -0.08 (-0.25 to 0.09)  In CSB: 0.12 (-0.06 to 0.29) |
| **Language domain** | 0.01 (-0.09 to 0.10) | 0.09 (-0.01 to 0.18) | 0.06 (-0.05 to 0.18) | 0.11 (0.003; 0.23) |

Data are mean difference (95% CI) based on intention-to-treat population. Linear mixed models adjusted for baseline measure of the outcome, WHZ, MUAC, HAZ, age, sex, month of inclusion (random effects: site). ITT = intention-to-treat. *Interaction between matrix and milk protein: p =0.098.

**Table E in S1 Table: Effects of supplementary foods on MDAT z-scores after intervention (24-wk, n=1,503): unadjusted analysis**

|  | **Matrix:**  **LNS versus CSB** | **Soy quality:**  **Isolate versus dehulled** | **Milk protein:** | |
| --- | --- | --- | --- | --- |
|  |  |  | **20% versus 0%** | **50% versus 0%** |
| **Gross motor domain** | -0.03 (-0.12; 0.06) | 0.02 (-0.06; 0.11) | -0.05 (-0.16; 0.06) | -0.05 (-0.15; 0.06) |
| **Fine motor domain** | -0.005 (-0.10; 0.09) | 0.04 (-0.05; 0.13) | 0.01 (-0.10; 0.12) | -0.04 (-0.15; 0.07) |
| **Language domain** | -0.14 (-0.27; 0.002) | 0.10 (-0.04; 0.24) | -0.06 (-0.23; 0.11) | -0.005 (-0.16; 0.17) |

Data are mean difference (95% CI) based on intention-to-treat population. Linear mixed models adjusted for baseline measure of the outcome (random effects: site and study id). ITT = intention to treat. There were no interactions between the 3 factors of food supplements (all p >0.43).

**Table F in S1 Table: Effects of supplementary foods on MDAT z-scores after intervention (24-wk) in PP population (n=1,382)**

|  | **Matrix:**  **LNS versus CSB** | **Soy quality:**  **Isolate versus dehulled** | **Milk protein:** | |
| --- | --- | --- | --- | --- |
|  |  |  | **20% versus 0%** | **50% versus 0%** |
| **Gross motor domain** | 0.02 (-0.06; 0.11) | -0.01 (-0.09; 0.08) | -0.04 (-0.15; 0.06) | -0.05 (-0.15; 0.06) |
| **Fine motor domain** | 0.04 (-0.05; 0.14) | 0.01 (-0.09; 0.10) | -0.004 (-0.12; 0.11) | -0.03 (-0.14; 0.09) |
| **Language domain** | -0.04 (-0.17; 0.09) | 0.04 (-0.09; 0.17) | -0.07 (-0.23; 0.08) | -0.01 (-0.17; 0.14) |

Data are mean difference (95% CI) based on intention-to-treat population. Linear mixed models adjusted for baseline measure of the outcome, WHZ, MUAC, HAZ, age, sex, and month of inclusion (random effects: site and id). PP = per protocol. There were no interactions between the 3 factors of food supplements (all p >0.34).
