# Supplementary material for: Impact of food supplements on early child development in children with moderate acute malnutrition: A randomised 2 x 2 x 3 factorial trial in Burkina Faso
Source: PLoS Med. 2020 Dec 23;17(12):e1003442. doi: 10.1371/journal.pmed.1003442 (PMC7757806; doi:10.1371/journal.pmed.1003442)
Supplement: Attachment — Submitted filename: 2x2x3 fact trial in BFA PMEDICINE D 20 02543R1.pdf [file pmed.1003442.s004.pdf]

Peer review: Effectiveness of food supplements on early child development in children with moderate acute malnutrition: a randomized 2*3*3 factorial trial in Burkina Faso

Introduction:

This is a trial comparing various standardized food supplements to children 6-23 months suffering from moderate- acute- malnutrition MAM, meaning they have weight-for-height under the “within-normal” threshold, less than z-score -2 according to the WHO Child Growth Standards. However, they have not yet reached the severe stage being below -3 or with other clinical signs of severity. MAM and SAM combined are called ‘wasting’ and part of our global nutritional targets is to reduce wasting to less than 5% by 2030. This aim allows twice as high wasting percentage than what a normal distribution of its “natural” healthy occurrence should allow (approx. 2.5% above and below +/- 2 z-scores from the reference median), so that aim isn’t too ambitious and given right investments and reduced inequities we could reach it. However, the world is off-track reaching that aim and is currently not doing enough for children being too thin. Below I paste in a figure displaying current global numbers from the last GNR.

Obviously, a better understanding of necessary preventive and treatment efforts, particularly involving food to children having experienced hunger or disease resulting in a wasting condition, are warranted. The current research is a major contribution to that understanding. As far as I have understood the research team has previously presented the outcome variables: hemoglobin, iron status, anthropometry, and inflammation (2018) and fat-free mass (2017) from the same intervention. The current paper presents child development indicators and both the trial and the child development outcomes serve as a ‘landmark’ paper and the work merits publication in a ‘flagship’-journal, in this case PlosMedicine. I also have the knowledge that PlosMedicine has taken responsibility for presenting global trends in this regard. However, before publication, I would recommend some substantial revision of the current presentation to fully inform the readers and contextualize the work and this group of children better.

Child (under-five) nutrition status

Coexistence of wasting, stunting and overweight

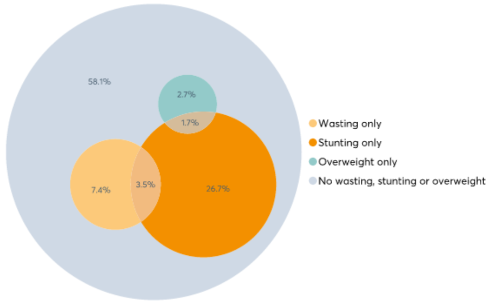

Sources: UNICEF, Division of Data Research and Policy (2019). UNICEF Global Databases: Overlapping Stunting, Wasting and Overweight, January 2019, New York.

Notes: Percentage of children under-five years of age who experience different and overlapping forms of malnutrition. Based on population weighted means of 111 countries.

Major comments:

My major questions that I am left with, which I did not at a glanse managed to get a full overview of after looking at the referenced papers (12,13,14), were regarding the context, design, intervention and outcome.

First, the context. I would appreciate that the paper followed a standard flow in the methods giving the reader a site description first. They clearly state the number of villages, that it was a humanitarian setting with a GAM >10%. In line with this they mention the nutritional recovery rates and main findings of the trial in the introduction without mentioning the numbers/figures. The introdction and metods would benefit from deeper and more specific explanation (12).

I am very sorry to admit that I am not knowledgeble about Province du Passoré even if I actually have been to BFA 2-3 times and worked closely with colleagues from that country. When that is my experience I would expect that a general international reader would know less than me. As a reader I would like to know how the region is doing compared to the rest of the country with respect to key indicators such as child mortality and the country. How long-standing had the GAM situation been at this level and was it related to conflict or weather disaster? What were the responses? Also, this being a 12 week intervention, how did that intervention collide with the seasonal variation that year? I was trying to search about Province du Passoré not to flash my ignorance here, but I did not get adequately informed. Kindly contextualise the research site better.

Second, design: The authors write quite a bit about the epidemiological design in terms of the trial design and sample size etc, but I am left with questions about the 'doing.' It seems like the study team has screened for MAM-children involving CHWs in the communities (143 villages) and at the 5 clinics. Was the aim to find "all" children with MAM in the study area until the sample size was reached? Was there any kind of 'random' inclusion into the study meaning that more children went into the feeding program for MAM than the ones that were enrolled in the study? Some more information about the recruitment, inclusion and sampling frame would ease understanding of the design.

Also, line 91 informs us that the children could be recruited based on caregivers initiative.
Was the trial known in the area?

Would the authors characterise the study as a community trial or a clinical trial?

Were all the assessments which went into the database done at the clinics? I am informed that there is a basline-endline (12 weeks) and post intervention (24 weeks). Were all these assessments, the lab, the questionnaire, anthropometry etc done at the clinic? How did they assure follow-up? I don't see this answered in the 2017-paper either, however, a bit of the measurments and randomisation were repeated across the papers. If this area had GAM rates > 10% I would assume that infrastructure etc was not so good? I would prefer that enough was explained to ease understanding answering the 'how'-questions in each individual paper.

Third, the intervention: The authors fully explain the supplements.

As they describe they get 500kcal/daily serving. Were any measures taken to evaluate the compliance/fidelity (meaning uptake) to this intervention? How was the intervention delivered? Could they, or were they adviced not to give other foods (except breastfeeding?)? I see an acceptability paper from 2016, but that is not informing me about the fidelity in the current study.

The lines 205-208 make me wonder more as a substantial group of children (n=102) develop SAM and it is mentioned that they received other food (which I guess here is therapautic food). This information, combined with the very low loss-to-follow-up, the relatively ‘high’ median of WHZ within the MAM group, make me wonder about details surrounding the frequency, delivery and measurement of the intervention.

Although the discussion mentions very many valid points, I miss after line 311 an eleaborated discussion on how breasteeding was in this study, how the findings could be related to a) the actual weight gain or b) reduced hunger in this patient group.

Regarding the MDAT outcome. Reference 18 (2020) refers to as far as I can see a presentation of mean scores in the same population and calculated correlates of the MDAT scores with other co-factors such as anthropometry and nutritional and inflammation markers in the same trial population.

Ref 17 refers to Gladstone’s and colleagues pioneer work in 2010 in Malawi developing and validating the MDAT. Most likely it is very acceptable with a transfer and adaptation of the tool within sub-Saharan Africa from Malawi to Burkina Faso, but it would be good if the processes regarding the tool use (translations, adaptations, any validation (?), norming (?)) was clearly described). If that was not done, in-country validation and norming, that should be stated. Also, what has happened to the tool after 2010 in terms of devlopment? Has MDAT been used in another BFA-nearby country? (The distance between Malawi and Burkina Faso is more than the distance from Burkina Faso to Copenhagen)

Ethics and authorship: What is a consultative approval obtained in Denmark?
Only 1/15 authors had an affiliation in Burkina Faso. Was ethical approval needed for any of the other researchers (Finland, UK, France?)?
Comment: I cannot help wondering why not more colleagues with institutional affiliation in Burkina Faso were more strongly represented in the author list. I’m just questoning if there may have been some exclusion of authors that were contributing or could have been contributing to the paper if included. This is obviously a very complex trial at the design, conduct, analysis and writing stage and more authors representing different within country roles would have been natural.

Minor:

Abbreviations: Generally check that all the abbreviations are spellout and put in parenthesis in the main manuscript first time they are mentioned. See e.g. SI and DS, line 103
I actually think this complex paper would benefit from an abbreviation list.

Rewrite: Line 84 “which were staffed” to e.g. “Health workers from …ALIMA, Senegal were working in the five health centres in the trial”
Also, provide a brief description of their qualifications (nurses, nutritionists, docotors, educational level ??)

In the analysis they talk about the site-specific random effects. Are the sites the “health-centres” or something else?

Table 2: Could 95% CI be presented with the means in all columns?

I am confused about the post-hoc sex analysis presented in the abstract and the statement in the conclusion that more sex-specific effect should be studied.

I would wonder whether fidelity, sustainability issues and further efforts to reduce SAM should also be highlighted?

Also in the abstract , I would prefer if the findings focused on the key outcomes per arm first rather than the post-hoc findings. Also, the findings could present the ‘recovery’ and SAM-statistics.

In conclusion:

This paper represents massive work from a distinguised group of reseerachers in nutrition, epidemiology, clinical and public health on one of the key issues under the SDG era, namely feeding and follow up of wasted children. The paper is in my understanding a land-mark paper that would benefit from editing making it a ‘stand-alone’ paper. So the authors should explain, present and discuss more, not less in order to inform the global debate on how to reach the target on reducing wasting.
