# Supplementary material for: Impact of food supplements on early child development in children with moderate acute malnutrition: A randomised 2 x 2 x 3 factorial trial in Burkina Faso
Source: PLoS Med. 2020 Dec 23;17(12):e1003442. doi: 10.1371/journal.pmed.1003442 (PMC7757806; doi:10.1371/journal.pmed.1003442)
Supplement: Attachment — Submitted filename: Response letter_Plos Med.docx [file pmed.1003442.s005.docx]

**Response letter for manuscript** **"Effectiveness of food supplements on early child development in children with moderate acute malnutrition: a randomized 2 x 2 x 3 factorial trial in Burkina Faso" (PMEDICINE-D-20-02543R1)**

Dear editor,

We thank you for reviewing our manuscript and considering it for publication in PLOS Medicine. Please find below our point-by-point responses to the comments made by the editors and the reviewers. All revisions have been tracked in the manuscript.

***Requests from the editors:***

**Per the journal's usual guidelines, we'd ask that the authors include in the Abstract Methods and Findings section a brief summary of any key limitation(s) of the study's methodology.*

Response:

We have revised the Abstract to include the study’s key limitations (line 43-44).

**At this stage, we ask that you include a short, non-technical Author Summary of your research to make findings accessible to a wide audience that includes both scientists and non-scientists. The Author Summary should immediately follow the Abstract in your revised manuscript. This text is subject to editorial change and should be distinct from the scientific abstract. Please see our author guidelines for more information:* [*https://eur02.safelinks.protection.outlook.com/?url=https%3A%2F%2Fjournals.plos.org%2Fplosmedicine%2Fs%2Frevising-your-manuscript%23loc-author-summary&amp;data=02%7C01%7Cmeo%40nexs.ku.dk%7Ca5e70f8c63534d93c78408d83302cec3%7Ca3927f91cda14696af898c9f1ceffa91%7C0%7C0%7C637315434447092675&amp;sdata=%2BZQgaQKRELQwW7zx%2BMxjhe%2FjTrc%2Bys6nWcghJIQd3nY%3D&amp;reserved=0*](https://eur02.safelinks.protection.outlook.com/?url=https%3A%2F%2Fjournals.plos.org%2Fplosmedicine%2Fs%2Frevising-your-manuscript%23loc-author-summary&amp;data=02%7C01%7Cmeo%40nexs.ku.dk%7Ca5e70f8c63534d93c78408d83302cec3%7Ca3927f91cda14696af898c9f1ceffa91%7C0%7C0%7C637315434447092675&amp;sdata=%2BZQgaQKRELQwW7zx%2BMxjhe%2FjTrc%2Bys6nWcghJIQd3nY%3D&amp;reserved=0)

Response:

We have included a non-technical Author Summary in the revised manuscript (line 50-79).

**It's not clear that the authors have fully used the CONSORT guidance in reporting the trial results - a CONSORT flow diagram is included (fig 2) which is great, but the full CONSORT guidance and checklist should also be used - and the completed checklist uploaded with the revised paper as supporting information. The authors can use the regular CONSORT instruments (*[*https://eur02.safelinks.protection.outlook.com/?url=https%3A%2F%2Fwww.equator-network.org%2Freporting-guidelines%2Fconsort%2F&amp;data=02%7C01%7Cmeo%40nexs.ku.dk%7Ca5e70f8c63534d93c78408d83302cec3%7Ca3927f91cda14696af898c9f1ceffa91%7C0%7C0%7C637315434447092675&amp;sdata=7BFJ%2Fo3q3MGpblv7%2BJaoc2nKnynyBp7xsjP6dBJWLqY%3D&amp;reserved=0*](https://eur02.safelinks.protection.outlook.com/?url=https%3A%2F%2Fwww.equator-network.org%2Freporting-guidelines%2Fconsort%2F&amp;data=02%7C01%7Cmeo%40nexs.ku.dk%7Ca5e70f8c63534d93c78408d83302cec3%7Ca3927f91cda14696af898c9f1ceffa91%7C0%7C0%7C637315434447092675&amp;sdata=7BFJ%2Fo3q3MGpblv7%2BJaoc2nKnynyBp7xsjP6dBJWLqY%3D&amp;reserved=0)*)*

*or the more recent specialist guideline for reporting multi-arm randomized trials (*[*https://eur02.safelinks.protection.outlook.com/?url=https%3A%2F%2Fjamanetwork.com%2Fjournals%2Fjama%2Ffullarticle%2F2731183&amp;data=02%7C01%7Cmeo%40nexs.ku.dk%7Ca5e70f8c63534d93c78408d83302cec3%7Ca3927f91cda14696af898c9f1ceffa91%7C0%7C0%7C637315434447092675&amp;sdata=dS4mw%2Bd1ZCkH3CKeQnZu050cB838mgvT9yQxdYaQZas%3D&amp;reserved=0*](https://eur02.safelinks.protection.outlook.com/?url=https%3A%2F%2Fjamanetwork.com%2Fjournals%2Fjama%2Ffullarticle%2F2731183&amp;data=02%7C01%7Cmeo%40nexs.ku.dk%7Ca5e70f8c63534d93c78408d83302cec3%7Ca3927f91cda14696af898c9f1ceffa91%7C0%7C0%7C637315434447092675&amp;sdata=dS4mw%2Bd1ZCkH3CKeQnZu050cB838mgvT9yQxdYaQZas%3D&amp;reserved=0)*).*

Response:

An updated CONSORT checklist has been added as supplementary information.

**Per journal policy a copy of the final trial protocol should be uploaded as supporting information with the revised paper, or alternatively if the protocol was published in a journal then the citation for that can be given (in which case no need to upload the file).*

Response:

The final trial protocol has been uploaded as supplementary information.

**In the paper, a number of post-hoc analyses are reported, eg those regarding improvement of language scores in boys only. It would be good to include some further discussion about the possible limitations of such post-hoc (presumably, not specified in the protocol) analyses, and also to what extent those findings are biologically plausible (which would strengthen the claims).*

Response:

In our analyses, we found that several of the effects of supplementation were modified by sex, i.e. the effect of milk protein on language z-scores and the effect of LNS over CSB products on gross motor z-scores were only seen in boys. Sex-specific effects are rarely investigated in supplementation trials and we believe it is a strength of our study, that we’ve analysed and reported these, although we agree it is a limitation that this subgroup analysis was not pre-specified in the protocol. In the revised version, we have emphasized the limitations of the post-hoc analyses (line 40 + 345-346 + 356-357) and added a discussion of possible explanations (line 395-402).

-----------------------------------------------------------

***Comments from the reviewers:***

***Reviewer #1:***

*Dear authors, kindly see my attached letter. Kindly keep up the good work. We as colleagues in this field want more information from this study.*

*Peer review: Effectiveness of food supplements on early child development in children with moderate acute malnutrition: a randomized 2*3*3 factorial trial in Burkina Faso*

*Introduction:*

*This is a trial comparing various standardized food supplements to children 6-23 months suffering from moderate- acute- malnutrition MAM, meaning they have weight-for-height under the “within-normal” threshold, less than z-score -2 according to the WHO Child Growth Standards. However, they have not yet reached the severe stage being below -3 or with other clinical signs of severity. MAM and SAM combined are called ‘wasting’ and part of our global nutritional targets is to reduce wasting to less than 5% by 2030. This aim allows twice as high wasting percentage than what a normal distribution of its “natural” healthy occurrence should allow (approx. 2.5% above and below +/- 2 z-scores from the reference median), so that aim isn’t too ambitious and given right investments and reduced inequities we could reach it. However, the world is off-track reaching that aim and is currently not doing enough for children being too thin. Below I paste in a figure displaying current global numbers from the last GNR.*

*Obviously, a better understanding of necessary preventive and treatment efforts, particularly involving food to children having experienced hunger or disease resulting in a wasting condition, are warranted. The current research is a major contribution to that understanding. As far as I have understood the research team has previously presented the outcome variables: hemoglobin, iron status, anthropometry, and inflammation (2018) and fat-free mass (2017) from the same intervention. The current paper presents child development indicators and both the trial and the child development outcomes serve as a ‘landmark’ paper and the work merits publication in a ‘flagship’-journal, in this case PlosMedicine. I also have the knowledge that PlosMedicine has taken responsibility for presenting global trends in this regard. However, before publication, I would recommend some substantial revision of the current presentation to fully inform the readers and contextualize the work and this group of children better.*

*Major comments:*

*My major questions that I am left with, which I did not at a glanse managed to get a full overview of after looking at the referenced papers (12,13,14), were regarding the context, design, intervention and outcome.*

*First, the context. I would appreciate that the paper followed a standard flow in the methods giving the reader a site description first. They clearly state the number of villages, that it was a humanitarian setting with a GAM >10%. In line with this they mention the nutritional recovery rates and main findings of the trial in the introduction without mentioning the numbers/figures. The introdction and metods would benefit from deeper and more specific explanation (12).*

Response:

The methods section now starts with a more detailed description of the study setting (line 122-139). We have added the overall recovery rate to the introduction (line 117-118). The specific numbers/figures for main findings of the intervention have been given in previous publications, which we refer to.

*Reviewer #1:*

*I am very sorry to admit that I am not knowledgeble about Province du Passoré even if I actually have been to BFA 2-3 times and worked closely with colleagues from that country. When that is my experience I would expect that a general international reader would know less than me. As a reader I would like to know how the region is doing compared to the rest of the country with respect to key indicators such as child mortality and the country. How long-standing had the GAM situation been at this level and was it related to conflict or weather disaster? What were the responses? Also, this being a 12 week intervention, how did that intervention collide with the seasonal variation that year? I was trying to search about Province du Passoré not to flash my ignorance here, but I did not get adequately informed. Kindly contextualise the research site better.*

Response:

We have elaborated on the description of the study setting and GAM situation to improve the contextualization of our findings (line 122-139). Regarding seasonal variation, the children were recruited throughout the year (September 2013 to August 2014). Models of supplement effects were adjusted for month of inclusion – as described in methods (line 258).

*Reviewer #1:*

*Second, design: The authors write quite a bit about the epidemiological design in terms of the trial design and sample size etc, but I am left with questions about the ‘doing.’ It seems like the study team has screened for MAM-children involving CHWs in the communities (143 villages) and at the 5 clinics. Was the aim to find “all” children with MAM in the study area until the sample size was reached? Was there any kind of ‘random’ inclusion into the study meaning that more children went into the feeding program for MAM than the ones that were enrolled in the study? Some more information about the recruitment, inclusion and sampling frame would ease understanding of the design.*

Response:

The MAM sites were set up purely for the study purpose and the aim was to find all children until sample size was achieved. There was no random selection from a wider programme. We have clarified that no other supplementation programme for MAM was functioning on a regular basis in the area at the time of the study (line 137-139). We also give more information about the recruitment of children in the revised manuscript (line 158-160).

*Reviewer #1:*

*Also, line 91 informs us that the children could be recruited based on caregivers initiative. Was the trial known in the area?*

Response:

Yes, the trial was known in the area. Community meetings were held in each location prior to the start of the study. Here the purpose and the procedures were explained to community leaders and other community members. This has been clarified in the revised manuscript (line 158-160).

*Reviewer #1:*

*Would the authors characterise the study as a community trial or a clinical trial?*

Response:

We note that these terms are used inconsistently in the literature and that no universal definition exists. A discussion among the co-authors showed differences in opinions. We have therefore chosen to define the trial as an effectiveness study (mentioned in line 1, 30, 59, 118, 142, 256 + further emphasized in line 369-374), which is in line with the wording used in previous publications from the trial.

*Reviewer #1:*

*Were all the assessments which went into the database done at the clinics? I am informed that there is a basline-endline (12 weeks) and post intervention (24 weeks). Were all these assessments, the lab, the questionnaire, anthropometry etc done at the clinic? How did they assure follow-up? I don’t see this answered in the 2017-paper either, however, a bit of the measurments and randomisation were repeated across the papers. If this area had GAM rates > 10% I would assume that infrastructure etc was not so good? I would prefer that enough was explained to ease understanding answering the ‘how’-questions in each individual paper.*

Response:

Anthropometry, child development and Hb were assessed at the sites, while CRP was analysed in a lab in Germany – as described in methods. We have added more details about incentives and procedures for follow-up of participants (line 168-171).

*Reviewer #1:*

*Third, the intervention: The authors fully explain the supplements.*

*As they describe they get 500kcal/daily serving. Were any measures taken to evaluate the compliance/fidelity (meaning uptake) to this intervention? How was the intervention delivered? Could they, or were they adviced not to give other foods (except breastfeeding?)? I see an acceptability paper from 2016, but that is not informing me about the fidelity in the current study.*

Response:

Acceptability and feeding behaviours of the supplements have been reported in three previous papers (Iuel-Brockdorff 2015, 2016 and 2017). We have added more details on the delivery of interventions and findings of these sub-studies in the revised manuscript (line 183-192).

*Reviewer #1:*

*The lines 205-208 make me wonder more as a substantial group of children (n=102) develop SAM and it is mentioned that they received other food (which I guess here is therapautic food). This information, combined with the very low loss-to-follow-up, the relatively ‘high’ median of WHZ within the MAM group, make me wonder about details surrounding the frequency, delivery and measurement of the intervention.*

Response:

Children who developed SAM during the intervention period were treated with ready-to-use therapeutic food (RUTF; Plumpy’Nut, Nutriset) instead of the experimental supplement – as described in Methods (line 195-197). A total of 102 children developed SAM during the trial (102/1609 = 6.3%), which is in line with the rates reported in a systematic review of MAM treatment (ref 10, Gera 2017, data from 5 studies). This has been added to the discussion (line 357-359). We have also added additional details on the delivery of the intervention and assessment of its use (line 183-192) in the revised manuscript.

*Reviewer #1:*

*Although the discussion mentions very many valid points, I miss after line 311 an eleaborated discussion on how breasteeding was in this study, how the findings could be related to a) the actual weight gain or b) reduced hunger in this patient group.*

Response:

We acknowledge the importance of breastfeeding in this age group but, as mentioned, we did not find any difference in the proportion of children breastfed between groups. Our trial was an effectiveness study, which did not allow us to conclude on the underlying mechanisms of the effects of supplementation. The effects on language and motor skills may be partly related to increased well-being related to less hunger - in any case an effect of providing a supplement. The use of supplementation is likely to be similar to the actual use in a nutritional programme and the observed effects are therefore very relevant for practice. We have added a discussion of these points to the strengths and limitation paragraph of the revised manuscript (line 367-374).

*Reviewer #1:*

*Regarding the MDAT outcome. Reference 18 (2020) refers to as far as I can see a presentation of mean scores in the same population and calculated correlates of the MDAT scores with other co-factors such as anthropometry and nutritional and inflammation markers in the same trial population.*

*Ref 17 refers to Gladstone’s and colleagues pioneer work in 2010 in Malawi developing and validating the MDAT. Most likely it is very acceptable with a transfer and adaptation of the tool within sub-Saharan Africa from Malawi to Burkina Faso, but it would be good if the processes regarding the tool use (translations, adaptations, any validation (?), norming (?)) was clearly described). If that was not done, in-country validation and norming, that should be stated. Also, what has happened to the tool after 2010 in terms of development? Has MDAT been used in another BFA-nearby country? (The distance between Malawi and Burkina Faso is more than the distance from Burkina Faso to Copenhagen)*

Response:

The translation, validation, adaption, and pilot testing of the MDAT tool in our study setting has been described in detail in a previous paper that we refer to (line 214-215). The MDAT tool was developed in Malawi in 2010 but has since then been used in settings across Sub-Saharan Africa.

*Reviewer #1:*

*Ethics and authorship: What is a consultative approval obtained in Denmark? Only 1/15 authors had an affiliation in Burkina Faso. Was ethical approval needed for any of the other researchers (Finland, UK, France?)? Comment: I cannot help wondering why not more colleagues with institutional affiliation in Burkina Faso were more strongly represented in the author list. I’m just questoning if there may have been some exclusion of authors that were contributing or could have been contributing to the paper if included. This is obviously a very complex trial at the design, conduct, analysis and writing stage and more authors representing different within country roles would have been natural.*

Response:

Ethical approval was only a requirement from the Ethics Committee for Health Research in Burkina Faso. We also obtained a consultative approval from the Danish National Committee on Biomedical Research Ethics. This is an optional approval, in which the Danish committee offers to review studies with involvement of Danish investigators, while formal approval is given from another country. The author list includes two co-investigators from Burkina Faso, Charles Yaméogo and Albertine Ouédraogo. We agree with the reviewer that more authors with affiliation to Burkina Faso would have been desirable. In all our projects, we focus on research capacity building. In the current study a local medical doctor was employed as trial manager and it was planned for him to undertake a PhD based on the study. Tragically, he died during an outbreak of dengue fever during the study.

*Reviewer #1:*

*Minor:*

*Abbreviations: Generally check that all the abbreviations are spellout and put in parenthesis in the main manuscript first time they are mentioned. See e.g. SI and DS, line 103 I actually think this complex paper would benefit from an abbreviation list.*

Response:

The abbreviations have been checked and a list of the abbreviations added (line 81-95). We have dropped SI and DS to limit the use of abbreviations.

*Reviewer #1:*

*Rewrite: Line 84 “which were staffed” to e.g. “Health workers from …ALIMA, Senegal were working in the five health centres in the trial” Also, provide a brief description of their qualifications (nurses, nutritionists, docotors, educational level ??)*

Response:

We have revised the mentioned sentence about study personnel and mention their qualifications in the “data collection” section (line 124-125 + 221-222 + 224).

*Reviewer #1:*

*In the analysis they talk about the site-specific random effects. Are the sites the “health-centres” or something else?*

Response:

Yes, the sites here refer to the five study sites (health centres), that the study was carried out in. This has been clarified in the revised manuscript (line 258-259).

*Reviewer #1:*

*Table 2: Could 95% CI be presented with the means in all columns?*

Response:

We have considered this suggestion carefully and discussed it with our statistician. However, we prefer to present the mean z-scores at the three time points with SDs, as these are needed for interpretation of the relative size of the changes presented in the last columns as well as the effects of supplements shown in the subsequent tables.

*Reviewer #1:*

*I am confused about the post-hoc sex analysis presented in the abstract and the statement in the conclusion that more sex-specific effect should be studied.*

Response:

We have added more discussion on sex-specific effects seen, including the possible biological explanation (line 395-402) and the limitation of these being assessed in post-hoc analyses (line 40 + 345-346 + 356-357).

*Reviewer #1:*

*I would wonder whether fidelity, sustainability issues and further efforts to reduce SAM should also be highlighted?*

Response:

We have elaborated the discussion on these issues in the revised manuscript (line 367-374).

*Reviewer #1:*

*Also in the abstract , I would prefer if the findings focused on the key outcomes per arm first rather than the post-hoc findings. Also, the findings could present the ‘recovery’ and SAM-statistics.*

Response:

We agree with the reviewer that findings of primary analyses should, in principle, be presented before findings of secondary or post-hoc analyses. However, in the interest of a clear presentation of results from this complex factorial trial, we believe that the reader is better helped with a presentation of all relevant effects by factor (i.e. milk/soy/matrix). In the revised abstract, we’ve highlighted which results are based on post-hoc analyses (line 40). The findings related to nutritional recovery and SAM statistics have been published in a previous paper (ref 12, Fabiansen, Plos Medicine 2017), which we refer to in our method section. We have added the recovery rate to the introduction (line 117-118) but we prefer not to include the findings on recovery and SAM in the abstract, as they have been published previously and are not directly related to the specific aim of this paper.

*Reviewer #1:*

*In conclusion:*

*This paper represents massive work from a distinguised group of reserachers in nutrition, epidemiology, clinical and public health on one of the key issues under the SDG era, namely feeding and follow up of wasted children. The paper is in my understanding a land-mark paper that would benefit from editing making it a ‘stand-alone’ paper. So the authors should explain, present and discuss more, not less in order to inform the global debate on how to reach the target on reducing wasting.*

-----------------------------------------------------------

***Reviewer #2: Alex McConnachie, Statistical Review***

*Olsen and colleagues report a secondary analysis from a 2x2x3 factorial trial of food supplementation in Burkina Faso. This review considers the statistical aspects of the paper.*

*These are generally good. The outcome variables are validated measures, expressed as z-scores. The basic analysis plan involves fitting appropriately-adjusted regression models with 3-way intervention effect interactions and applying a backward selection procedure. The main effects and intervention interactions are reported clearly.*

*Subgroup analyses were applied to a number of baseline factors, though only interactions with sex are reported in the text, and in footnotes to the tables. None of the other factors indicated any intervention effect heterogeneity. Given that only sex showed any sign of interaction with the interventions, would a forest plot be a good way to present these more systematically?*

Response:

We note the approval of our analysis and report of findings. We appreciate the suggestion of presenting sex-specific effects in a forest plots, which we have discussed with our senior statistician. However, since these are based on post-hoc subgroup analyses and only pertain to a few of many comparisons, we do not find that presenting results by sex in a forest plot would benefit the reader’s overview of the study findings.

-----------------------------------------------------------

***Reviewer #3:***

*The team from Burkina Faso continues their series of papers analyzing a very impressive set of data and findings from their 2x2x3 factorial RCT of a variety of different supplementary foods for MAM. This study has been the source of several high-profile papers in the malnutrition community. Not surprisingly, given this experience, the manuscript is very well written and presented; the methods are tight and the results are well-analyzed. I usually have many many comments when reviewing manuscripts, but here the authors have delivered an excellently written manuscript.*

*Here, they focus on the extremely important outcomes of developmental milestones among survivors. The authors are to be congratulated for their attention to cognitive and physical development, something that is all too often lost in our rush to just focus on survival and nutritional* *recovery. As they are implicitly (and maybe even explicitly) emphasizing, it is just as important to survive well as to survive at all.*

*It is reassuring (in terms of internal face validity) to see that there is some dose-response relationship between the percentage of milk protein consumed and developmental scores.*

*Any explanation for the sex-specific effects seen?*

Response:

We have added a discussion on the possible biological explanation of the sex-specific effects seen in the study (line 395-402).

*Reviewer #3:*

*Some exploration of cost-benefit analysis would be helpful as well.*

*It would be fascinating in future research to evaluate these same developmental outcomes in trials where specific supplementation is not provided -- for example, those that are trialing direct cash transfer or counseling interventions.*

Response:

We agree with the reviewer that cost-benefit considerations are important to discussions about programs to prevent and/or treat acute malnutrition. However, a cost-benefit analysis was not part of the TreatFood trial.
