# Supplementary material for: Impact of food supplements on early child development in children with moderate acute malnutrition: A randomised 2 x 2 x 3 factorial trial in Burkina Faso
Source: PLoS Med. 2020 Dec 23;17(12):e1003442. doi: 10.1371/journal.pmed.1003442 (PMC7757806; doi:10.1371/journal.pmed.1003442)
Supplement: Attachment — Submitted filename: Comments to Effectiveness of food supplements on early child development in children with moderate acute malnutrition_ Plos medicine 2020 2nd review.docx [file pmed.1003442.s006.docx]

Comments to Effectiveness of food supplements on early child development in children with moderate acute malnutrition: a randomized 2 x 2 x 3 factorial trial in Burkina Faso

Thanks for the response letter and this revision. Things are well explained, the methods much clearer and the discussion interesting.

Almost all my comments are minor, however, I do think the document needs language polishing.

It’s technically correct, but sometimes grammar or language flow issues make reading heavier. Sometimes it looks like a few prepositions, articles etc have been cut to benefit the word count – but that may not benefit the reading. Also typography – included space before brackets etc.

I will first give my minor comments, then suggest something for the results and the discussion.

Minor:

Abstract: Methods and findings: 1^st^ sentence – the design mentioned followed by full stop, but the sentence is not a full sentence. E.g this was a ... trial …

L34: After presenting the trial arms before presenting the results, mention that differences in z-scores are presented by study arm and sex stratified

L36: … after **the** intervention, mean MDAT

L36-37: mention if this includes all study arms e.g. by including e.g. ‘overall’

After **the** intervention, the **overall** mean MDAT z-scores ….

L66: suggestons: … after the supplementation had ended..

L67: new bullet point for the sentence: “supplements containing milk…”

101: Rewrite to: An improved understanding of the plasticity of the brain **on top of a humanitarian obligation highlight** the importance of treating acute malnutrition and improve…

113: … to assess effects of **various types of food supplements**, the ….

150: It is a strange statement regarding the interaction and sample size. As long as interactions are not incorporated into the sample size estimation stratification would always reduce ‘power’ – by definition stratification is cutting the whole population in sub-groups which is in a way what one is doing with interaction.

226: wooden length board

**Question**: As these are small kids (<2 years) shouldn’t the entire document consider length rather than height, reflected as length boards, WLZ, LAZ etc? As you use WHO’s STATA package – that is the default.

235: …coefficients of variation (CVs) were <10%

240: I have been informed by our statistician that +/- SD is not a favored term, rather use: mean, SD

It is the calculations with SD that is adding or subtracting SD in a function.

246: It is not clear what: “these calculated values” refer to (is that “the mean of their last six items” or those failing six consecutive items?)

254: As you mention ITT – what do you do with the misses after randomization in analysis? Please describe – has relevance in the result section

290: What is meant by completed MDAT data? Should it be MDAT data collection?/tests?/assessment?

305 etc: either mean, SD or mean 95% CI

389: What are the numbers referring to?

424: I’ve commented on language before… …fewer children…

**To results:**

Would it be nice to display the 3 domains (motor, language and cognitive) in 3 figures and the key comparison arms presented in the results a BL – 12w and 24w showing the improvement in z-scores?

That should probably be displayed as refined version of this very simple old figure giving the z-scores on the y-axis and the time-points on the x-axis. From what you are writing be visualized whether there is progress-flattening after 12 weeks (or is it just the difference that disappeares). A figure would also ease reading.

<https://bmcpublichealth.biomedcentral.com/articles/10.1186/1471-2458-8-418/figures/2>

TRIAL FIGURE:

Would it be possible to display a branched profile? That would in a way be like pivot shifting Figure 1 90 degrees and adding numbers

**Discussion**

What is written from L463 and onwards is very well written. Keep.
