# Supplementary material for: Impact of food supplements on early child development in children with moderate acute malnutrition: A randomised 2 x 2 x 3 factorial trial in Burkina Faso
Source: PLoS Med. 2020 Dec 23;17(12):e1003442. doi: 10.1371/journal.pmed.1003442 (PMC7757806; doi:10.1371/journal.pmed.1003442)
Supplement: Attachment — Submitted filename: Response letter_ PMEDICINE-D-20-02543R2.2.docx [file pmed.1003442.s007.docx]

**Response letter for manuscript "Effectiveness of food supplements on early child development in children with moderate acute malnutrition: a randomized 2 x 2 x 3 factorial trial in Burkina Faso" (PMEDICINE-D-20-02543R2)**

Dear Editor,

We thank you again for reviewing our manuscript and for planning to accept it for publication in PLOS Medicine, provided the remaining editorial and production issues are dealt with.

Please find below our point-by-point responses to the comments made by the editors and the reviewers. The line numbers refer to where the revised text starts in the marked version of the manuscript.

------------------------------------------------------------

**Requests from Editors:**

1.	Please implement the final reviewer comments.

2.	Please update the title to: “Impact of food supplements on early child development in children with moderate acute malnutrition in Burkina Faso: neuropsychiatric outcomes from a randomized 2 x 2 x 3 factorial trial”.

Response: We have revised the first part of the title (Impact of…), but not the last part, since we don’t report neuropsychiatric outcomes. The outcomes are gross motor, fine motor and language development and we believe this is captured by the “early child development” in the title.

3.	The Data Availability Statement (DAS) requires revision. If the data are not freely available, please include an appropriate contact (web or email address) for inquiries (this cannot be a study author).

Response: The DAS states that the Danish Act on Data Protection does not allow for personal data to be made available to others without prior individual approval from the Danish Data Protection Agency. The revised DAS includes a website address with further information about the requirements and contact information for the Data Protection Officer of University of Copenhagen, who can be contacted about data inquiries.

4.	Abstract:

a.	Around lines 31-32, please include trial recruitment dates, and mention that it “took place at 5 health centres in Province du Passoré, Northern Region”.

Response: We have revised added more information, but left out the region, to be able to adhere to the maximum allowed word count of the abstract (line 30)

b.	Please state whether or not there was any blinding of participants or staff.

Response: We have included the information about blinding in the abstract (line 32) and also revised it in the method section (line 203) to clarify which groups were blinded.

c.	Around lines 33-34, please state the outcomes as follows: “This analysis presents secondary outcomes from the trial, which were…”.

Response: We have revised according to the suggestion (line 33)

d.	Around line 35, please give the age and sex demographics of the participants. Please also state how many were randomized to each group, and how many completed treatment in each group.

Response: We have added age and sex demographics to the abstract. We agree that it is good practice to report the number randomized and the number who completed the study in each group. However, as this factorial study design has 3 comparisons and a total of 12 groups, we think this data is much better presented in the flow chart. In the abstract we have included that the numbers randomized and completed were similar between groups (line 35)

e.	Line 36: Change to: “At 12 weeks, mean MDAT z-scores in the whole trial cohort had increased…”

Response: We have revised according to the suggestion, but left out “trial” to be able to adhere to the maximum allowed word count of the abstract (line 39)

f.	Line 38: We note that the 20% milk data are not statistically significant. Please reword to say that 50% was significant while 20% was not.

Response: We have reworded the text to the following:

Children had larger improvement of language z-scores if receiving supplements with milk (20%: 0.09 [-0.01, 0.19], p=0.08 and 50%: 0.11 [0.01, 0.21], p=0.02), although the difference only reached statistical significance for 50% milk. (line 43)

g.	Lines 39-40: Please reword to “Fine motor z scores were improved in participants receiving ...”

Response: We have reworded the text to the following:

The fine motor z-scores were also improved in children receiving milk, but only when 20% milk was added to CSB (0.18 [0.03, 0.33], p=0.02). (line 47)

h.	Line 41: Please avoid the term “tended”; instead please simple state whether this difference is statistically significant or not.

Response: We have reworded the text to the following:

Soy isolate over dehulled soy increased language z-scores by 0.07 (-0.01, 0.15), p=0.02, although the difference was not statistically significant. (line 46)

i.	Please quantify all results with p values and 95% CIs, including eg. lines 42-44.

Response: Both p-values and 95% Cis have been included in the revised abstract

j.	Please use square brackets when nesting, eg. “…(…[…]…)…”

Response: We have revised use of brackets according to this suggestion.

k.	Please mention whether or not any adverse events were recorded, and whether they were attributed to treatment.

Response: We have added text about adverse events to the abstract (line 37)

l.	Line 46: Please begin with “In this study, we found that…”

Response: We have revised according to the suggestion (line 52)

m.	Please include the trial registration number and primary source(s) of funding at the end.

Response: We have added information about trial registration and funding (line 57)

5.	Author Summary:

a.	Line 59: Please clarify “factorial” to a lay reader, or rephrase more simply.

Response: We agree that factorial is not a lay term. We have deleted it and think the sentence explains the design without use of this term (line 74)

b.	Line 63: Please clarify “cognitive z-scores” to a lay reader, or rephrase more simply.

Response: We have rephrased to describe the findings in lay terms (line 78+80).

6.	Please leave a space between text and citations, eg: “…cognitive skills in children[1] and the…”

Response: We have edited this throughout the manuscript.

7.	Line 124: Please include trial recruitment dates.

Response: We have added “in Burkina Faso in 2013-2014” to the introduction (line 115) and give the details of setting and date in the methods.

8.	Line 166: Please confirm whether consent was “informed”.

Response: We have added “informed” to the description of consent (line 283)

9.	Results: Please present the safety data for the study including numbers of specific events and whether or not adverse events are thought to be related to treatment.

Response: We have added this information to the revised manuscript (line 300)

10.	Lines 316, 323, 326, 437: Please replace “marginally significant”/”marginally associated” with “not significant”/”not associated”.

Response: We have reworded the text as follows:

Supplements with 20% milk protein had a marginally significant effect of 0.09 (95% CI: -0.01, 0.19) z-score, while supplements with 50% milk protein had an effect of 0.11 (95% CI: 0.01, 0.21) greater language z-score compared to supplements with 0% milk protein

->

Supplements with 20% milk protein increased the mean z-score by 0.09 (95% CI: -0.01, 0.19), although this was not statistically significant, while supplements with 50% milk protein increased the z-score by 0.11 (95% CI: 0.01, 0.21) compared to supplements with 0% milk protein

A milk protein content of 20% was also associated with improved fine motor z-scores, but only when added to CSB (0.18; 95% CI: 0.03, 0.33), while in LNS, it was marginally associated with lower fine motor z-scores (-0.14; 95% CI: -0.29, 0.01).

->

A milk protein content of 20% also increased fine motor z-scores, but only when added to CSB (0.18; 95% CI: 0.03, 0.33). In LNS, the fine motor z-scores were 0.14 lower (95% CI: -0.29, 0.01) in children receiving 20% milk protein compared to no milk protein, but this difference was not statistically significant.

Compared to dehulled soy, supplements with soy isolate were associated with a marginally significant 0.07 (95% CI: -0.01, 0.15) increase of language z-score.

->

Compared to dehulled soy, supplements with soy isolate increased language z-scores by 0.07 (95% CI: -0.01, 0.15), although this was not statistically significant.

Thus, it might be speculated whether isoflavones could explain both milk effects and the (marginally significant) benefit of soy isolate over dehulled soy.

->

Thus, it might be speculated whether isoflavones could explain both milk effects and the (statistically insignificant) benefit of soy isolate over dehulled soy.

11.	Lines 346-347: Please reword to: “…appeared to be more effective…”

Response: We have revised the text as suggested (line 371)

12.	Line 348: Please avoid the term “tendency”; instead please simple state whether this difference is statistically significant or not.

Response: We have reworded the text as follows:

We also found a tendency for soy isolate over dehulled soy to benefit language development.

->

We also found that soy isolate increased language z-score compared to dehulled soy, although the difference was not statistically significant

13.	Page 21: Please remove all sections here except the Acknowledgements.

Response: We have removed the other sections from the last page of the manuscript

14.	Please provide a DOI or URL for references 14, 15, and 24.

Response: We have added these details to the relevant references

15.	Please remove “Lond Engl.” from references 1 and 2.

Response: We have removed this detail from the relevant references

16.	When completing the CONSORT checklist, please use section and paragraph numbers, rather than page numbers.

Response: We have uploaded a revised CONSORT checklist with section and paragraph numbers

------------------------------------------------------------

**Comments from Reviewers:**

**Reviewer #1:**

*Dear authors, well done, I have some suggestions which I add in a separate letter, regards, Ingunn*

**Comments to Effectiveness of food supplements on early child development in children with moderate acute malnutrition: a randomized 2 x 2 x 3 factorial trial in Burkina Faso*

*Thanks for the response letter and this revision. Things are well explained, the methods much clearer and the discussion interesting.*

*Almost all my comments are minor, however, I do think the document needs language polishing.*

*It’s technically correct, but sometimes grammar or language flow issues make reading heavier. Sometimes it looks like a few prepositions, articles etc have been cut to benefit the word count – but that may not benefit the reading. Also typography – included space before brackets etc.*

*I will first give my minor comments, then suggest something for the results and the discussion.*

Response: We thank the reviewer for the overall positive comments on the last revisions made. We have made an effort to improve grammar and language flow of the second revision. A native English speaker has gone through it and made minor changes. We have also removed all spaces before brackets. These minor corrections are not marked in the revised manuscript.

**Minor:*

*Abstract: Methods and findings: 1^st^ sentence – the design mentioned followed by full stop, but the sentence is not a full sentence. E.g this was a ... trial …*

Response: We have revised the first sentence, so that it is now a full sentence: “We conducted a randomized…” (line 28)

**L34: After presenting the trial arms before presenting the results, mention that differences in z-scores are presented by study arm and sex stratified*

Response: Please note that both the editor and the reviewer have asked us to provide a lot of additional information to the abstract, which was already near the maximum allowed word count for abstracts in PLOS Medicine. We have managed to incorporate all of the other requests, but we find that this specific request would not improve the abstract, since it seems to communicate that all analyses are sex stratified, which was not the case. Instead, analyses by sex was a post-hoc subgroup analysis, as is clearly indicated when presenting the results in the abstract. The analytical approach is described in detail in the method section of the paper.

**L36: … after* ***the*** *intervention, mean MDAT*

Response: In response to a comment from the editor, we have revised this sentence to now read “At 12 weeks, the mean MDAT…” (line 39)

**L36-37: mention if this includes all study arms e.g. by including e.g. ‘overall’*

*After* ***the*** *intervention, the* ***overall*** *mean MDAT z-scores ….*

Response: In response to a comment from the editor, we have revised this sentence to now read “At 12 weeks, the mean MDAT z-scores in the whole cohort had increased by…” (line 39)

**L66: suggestons: … after the supplementation had ended..*

Response: We have revised according to the suggestion (line 82)

**L67: new bullet point for the sentence: “supplements containing milk…”*

Response: We have revised according to the suggestion (line 83)

**101: Rewrite to: An improved understanding of the plasticity of the brain* ***on top of a humanitarian obligation highlight*** *the importance of treating acute malnutrition and improve…*

Response: We have revised according to the suggestion (line 102)

**113: … to assess effects of* ***various types of food supplements****, the ….*

Response: We have revised the sentence to: *… to assess effects of* ***two types of food supplements*** *….* (line 115)

**150: It is a strange statement regarding the interaction and sample size. As long as interactions are not incorporated into the sample size estimation stratification would always reduce ‘power’ – by definition stratification is cutting the whole population in sub-groups which is in a way what one is doing with interaction.*

Response: The potential interactions between interventional factors were in fact incorporated into the sample size estimation. We have revised the description of sample size to clarify this (line 149).

**226: wooden length board*

Response: We have revised accordingly (line 228)

****Question****: As these are small kids (<2 years) shouldn’t the entire document consider length rather than height, reflected as length boards, WLZ, LAZ etc? As you use WHO’s STATA package – that is the default.*

Response: We agree that those are the standards. In this study, it is not very clear which term to use, since some of the children are older than 2 years at follow-up. However, we have changed height to length throughout the document since the majority of children are still <2 years of age.

**235: …coefficients of variation (CVs) were <10%*

Response: We have revised according to the suggestion (line 237)

**240: I have been informed by our statistician that +/- SD is not a favored term, rather use: mean, SD*

*It is the calculations with SD that is adding or subtracting SD in a function.*

Response: We have implemented this change in the revised manuscript and tables

**246: It is not clear what: “these calculated values” refer to (is that “the mean of their last six items” or those failing six consecutive items?)*

Response: It refers to data, where the mean of the last six items were used for imputations. We have clarified this in the revised text (line 247)

**254: As you mention ITT – what do you do with the misses after randomization in analysis? Please describe – has relevance in the result section*

Response: The main analyses are ITT based on complete cases. We have clarified this in the revised manuscript. The missing outcomes are not likely to cause risk of bias, since there are only few participants lost-to-follow-up (3.8% for the primary analysis at 12 weeks) and the frequency and causes are similar between treatment groups.

**290: What is meant by completed MDAT data? Should it be MDAT data collection?/tests?/assessment? -?*

Response: We write “All of the remaining 1,548 (96.2%) children had complete MDAT data and were included…” (i.e. not “completed MDAT data”) meaning that there was no missing MDAT data for these children (line 296)

**305 etc: either mean, SD or mean 95% CI*

Response: We present the MDAT z-scores with SDs for the descriptive estimates and with 95% CIs for the estimates of treatment effects.

**389: What are the numbers referring to?*

Response: The numbers refer to pooled effect estimates of nutritional supplements on development scores. We have clarified this in the revised manuscript (line 414).

**424: I’ve commented on language before… …fewer children…*

Response: We have revised this sentence as suggested (line 451) and made efforts to improve language throughout the manuscript.

****To results:***

*Would it be nice to display the 3 domains (motor, language and cognitive) in 3 figures and the key comparison arms presented in the results a BL – 12w and 24w showing the improvement in z-scores?*

*That should probably be displayed as refined version of this very simple old figure giving the z-scores on the y-axis and the time-points on the x-axis. From what you are writing be visualized whether there is progress-flattening after 12 weeks (or is it just the difference that disappeares). A figure would also ease reading.*

[*https://bmcpublichealth.biomedcentral.com/articles/10.1186/1471-2458-8-418/figures/2*](https://bmcpublichealth.biomedcentral.com/articles/10.1186/1471-2458-8-418/figures/2)

Response: We appreciate this suggestion and we agree that it would indeed be nice to include a visual presentation of the findings. However, the presentation of findings of this trial is complicated by the factorial design. The data would need to be presented by outcome (3 developmental domains) as well as by experimental factor (matrix, soy, milk), which is not easily illustrated in a figure, since there are interactions in effects between factors (in addition to sex-specific effects) for some but not all time points. Consequently, we prefer to keep the presentation of the data in tables.

**TRIAL FIGURE:*

*Would it be possible to display a branched profile? That would in a way be like pivot shifting Figure 1 90 degrees and adding numbers*

Response: We acknowledge the idea of including a branched profile showing numbers in each of the 12 cells. However, the most relevant comparisons in the factorial design are the pairwise comparisons by trial arm and we find that an overview of the study design and participants in each group is better presented in the current Fig 2 (the flow chart showing the number of participants by trial arm) in combination with the current figure 1 (showing all combinations of the 3 experimental factors).

****Discussion***

*What is written from L463 and onwards is very well written. Keep.*

------------------------------------------------------------

**Reviewer #3:**

**The authors have satisfactorily answered my queries.*

------------------------------------------------------------
